# Defining End-of-Life Preparedness: A Dyadic Qualitative Study of Hispanic Patients with Advanced Breast Cancer and Their Caregivers

**DOI:** 10.3390/healthcare14162471

**Published:** 2026-08-10

**Authors:** Lianel P. Rosario-Ramos, Carolina Quiles-Bengochea, Guillermo Laporte-Estela, Nashali Rivera, Angelique M. Graulau-Burgos, Cristina Peña-Vargas, Ruthmarie Hernández-Torres, Cynthia Cortes-Castro, Zindie Rodriguez-Castro, Eida M. Castro-Figueroa, Normarie Torres-Blasco

**Affiliations:** 1 School of Behavioral and Brain Sciences, Ponce Health Sciences University, Ponce, PR 00717, USA; glaporte22@stu.psm.edu (G.L.-E.); agraulau24@stu.psm.edu (A.M.G.-B.); cpena@psm.edu (C.P.-V.); ecastro@psm.edu (E.M.C.-F.);; 2 Ponce Research Institute, Ponce, PR 00717, USAccortes@psm.edu (C.C.-C.); zrodriguez@psm.edu (Z.R.-C.); 3 Memorial Sloan Kettering Cancer Center, New York, NY 10065, USA

**Keywords:** end-of-life preparedness, advanced breast cancer, Hispanic, patient–caregiver dyad, qualitative, Dyadic Cancer Outcomes Framework, health disparities, spiritual preparedness

## Abstract

**Background/Objectives**: End-of-life (EOL) preparedness remains critically understudied among Hispanic patients with advanced breast cancer and their patient–caregiver dyads, despite evidence that preparedness significantly influences quality of life, care decisions, and caregiver well-being. This study aimed to explore how Hispanic patient–caregiver dyads conceptualize and experience EOL preparedness. **Methods:** A qualitative descriptive design was employed, guided by the Dyadic Cancer Outcomes Framework, which highlights patient and caregiver characteristics, relationship processes, individual and relational outcomes, the cancer care trajectory, and the broader social context as interrelated influences on dyadic experience. Semi-structured individual interviews were conducted in Spanish with 11 metastatic patient–caregiver dyads (n = 22 participants) recruited through Ponce Health Sciences University and the Ponce Research Institute in Puerto Rico. Data were analyzed using codebook thematic analysis in NVivo 15, with themes interpreted through the framework’s components. **Results:** Six interdependent themes of EOL preparedness were identified: psychological and emotional, spiritual, informational, practical, physical, and caregiver role preparedness. Spiritual preparedness, grounded in faith, prayer, and surrender to divine will, functioned as the foundational axis organizing all other themes. Preparedness was dynamic and turning-point-driven, challenged anew at each stage of disease progression. Financial vulnerability, caregiver invisibility within formal care systems, and insufficient anticipatory information were identified as primary barriers. Family support and faith communities were the most consistently cited facilitators. **Conclusions**: The findings yield the first grounded, dyadic conceptualization of EOL preparedness with Hispanic advanced breast cancer patient–caregiver dyads. We propose a formal definition positioning preparedness as a dynamic, multidimensional, relationally embedded, and spiritually anchored process that is fundamentally interdependent between patient and caregiver. These results directly inform the development of a culturally tailored, dyadic EOL preparedness intervention for this underserved population.

## 1. Introduction

Cancer represents a critical public health challenge among Hispanic populations [1]. Breast cancer is the most frequently diagnosed malignant neoplasm among women in Puerto Rico, representing 30.5% of all new cases between 2016 and 2020, and the leading cause of cancer death in women on the island [2]. This burden is compounded by a high prevalence of late-stage diagnoses: Hispanic women are less likely to be diagnosed at a localized stage (60%) compared to non-Hispanic White women (68%), reducing the five-year relative survival rate in Puerto Rico to 31.5% at the metastatic stage [1,2].

Advanced breast cancer triggers a multidimensional crisis affecting patients and their families simultaneously. Patients face a substantial physical symptom burden, high rates of clinical anxiety and depression (35% to 50%), and severe financial toxicity, challenges that are intensified in Puerto Rico by structural poverty affecting 41.7% of the population, specialist shortages, and insufficient access to hospice services [3,4,5,6,7]. These adversities do not progress in isolation. The cultural value of *familismo* places caregiving almost exclusively on the family unit, and psychological distress operates bidirectionally within the patient–caregiver dyad: the suffering of one intensifies the distress of the other [8,9].

Preparedness has emerged as a critical determinant of quality of life and quality of dying in advanced cancer [9,10]. It is conceptualized as a dynamic, multidimensional state of readiness that enables patients and caregivers to anticipate and respond to the progressive demands of terminal illness, encompassing practical care planning, emotional adjustment, open communication about end-of-life wishes, complex decision-making, and the capacity to adapt as illness evolves [9,11]. From the patient’s perspective, end-of-life aspects involve advance care planning, symptom management, spiritual and emotional well-being, relationship adjustment, financial preparation, and the preservation of autonomy in care decisions [10,12]. For caregivers, it involves accessing resources, navigating healthcare systems, understanding treatments, providing support, and managing the tension between confidence in their caregiving role and the burden it carries [10,13,14].

Despite this evidence, end-of-life components have largely been studied in a fragmented manner, focusing separately on patients or caregivers rather than the relational unit [10,12]. Existing definitions treat preparedness primarily as the caregiver’s individual readiness to manage tasks, rather than a dyadic component [15,16], failing to accommodate the relational, spiritual, and culturally situated themes central to Hispanic dyads. Critically, no study to date has proposed or empirically examined a dyadic conceptualization of EOL preparedness for this population. A dyadic, culturally grounded conceptualization is needed.

The Dyadic Cancer Outcomes Framework [17] offers the appropriate conceptual lens to address this gap. It positions the patient–caregiver dyad as the unit of analysis, accommodates diverse relationship types beyond spousal pairs, and centers social and contextual factors as constitutive of outcomes rather than peripheral influences. Guided by this framework, the present study aims to describe how Hispanic advanced breast cancer patient–caregiver dyads conceptualize and experience EOL preparedness and, for the first time, propose an empirically grounded dyadic definition of EOL preparedness while situated within the lived experience of this population.

## 2. Materials and Methods

### 2.1. Study Design

This study employed a qualitative descriptive design using semi-structured individual interviews. Qualitative methods were selected because they are well-suited to the study’s primary aim: to explore and conceptualize the lived experience of EOL preparedness among Hispanic advanced breast cancer patient–caregiver dyads, a phenomenon that has not been previously examined from a dyadic perspective and for which quantitative measurement instruments do not yet exist. A descriptive qualitative approach allowed participants to articulate their experiences, needs, and understanding of preparedness in their own terms, generating the empirical foundation necessary for conceptual development and future intervention design [18].

The Dyadic Cancer Outcomes Framework [17] served as the guiding conceptual framework. This framework was selected because it explicitly positions the patient–caregiver dyad as the unit of analysis, accommodates diverse relationship types beyond spousal dyads, and foregrounds social and contextual factors as constitutive of outcomes. The framework comprises several interrelated components: patient and caregiver characteristics (including demographic, psychosocial, and cancer-related factors); relationship characteristics (type, quality, duration, and proximity); relationship processes (social support, coping strategies, communication, health behaviors, and physical caregiving tasks); individual patient outcomes, individual caregiver outcomes, and relationship-level outcomes; the cancer care trajectory across diagnosis, treatment, and survivorship; and the broader social context, including health policy and social determinants of health. These components informed the development of the interview guide and the organization of the codebook thematic analysis.

### 2.2. Setting

The study was conducted at Ponce Health Sciences University (PHSU) and the Ponce Research Institute (PRI), located in Ponce, Puerto Rico.

### 2.3. Participants and Recruitment

Participants were recruited from August to December of 2026 through multiple channels, including provider referrals, intervention flyers posted at the PHSU/PRI oncology clinic and community centers, social media outreach, and recruitment tables at community cancer support group events. Additionally, they were recruited through the Integrated Psychosocial Support Program (PAPSI), an evidence-based psychosocial support program integrated within the oncology care structure at PHSU/PRI. Dyads were the unit of enrollment. Each dyad consisted of one patient and one caregiver, with caregivers identified by the patient. To be eligible, patients were required to self-identify as Hispanic, be 21 or older, and have a confirmed diagnosis of Stage III or Stage IV breast cancer. Informal caregivers were required to self-identify as Hispanic, be over 21 years of age, and be identified by the patient as their primary informal caregiver. Caregivers were not required to be spouses or co-residents and included mothers, daughters, siblings, husbands, and other family members. Participants were excluded if they had a disabling medical or psychiatric condition, were unable to understand the consent procedure, or were too medically ill to participate. A total of 11 patient–caregiver dyads (N = 22 participants) were enrolled and completed interviews. Patient–caregiver dyads were compensated for their time and received $20 USD each post-interview.

### 2.4. Data Collection

Data were collected through hour-long semi-structured individual interviews (Appendix A) conducted in Spanish via phone call. A semi-structured interview guide was developed by the research team (L.R.R. and N.T.B.) and informed by a systematic review of the EOL preparedness literature and the components of the Dyadic Cancer Outcomes Framework [17]; the interview guide did not go through a pilot study evaluation. The interview guide covered participants’ personal definitions of preparedness; diagnosis experience and initial sense of preparedness; emotional, physical, practical, spiritual, and informational themes of preparedness over time; relational processes within the dyad; cultural and spiritual factors; financial concerns; advance care planning; social support and unmet needs; and suggestions for future intervention. Patients and caregivers were interviewed separately to allow free expression. Two dyads elected to be interviewed jointly; this was honored and noted in the analysis. Interviews were audio-recorded with participant permission, transcribed verbatim by the first author, and reviewed for accuracy prior to analysis. All interviews were conducted in Spanish by L.R.R. and are reported in English translation throughout this manuscript.

### 2.5. Data Analysis

**Figure 1:**
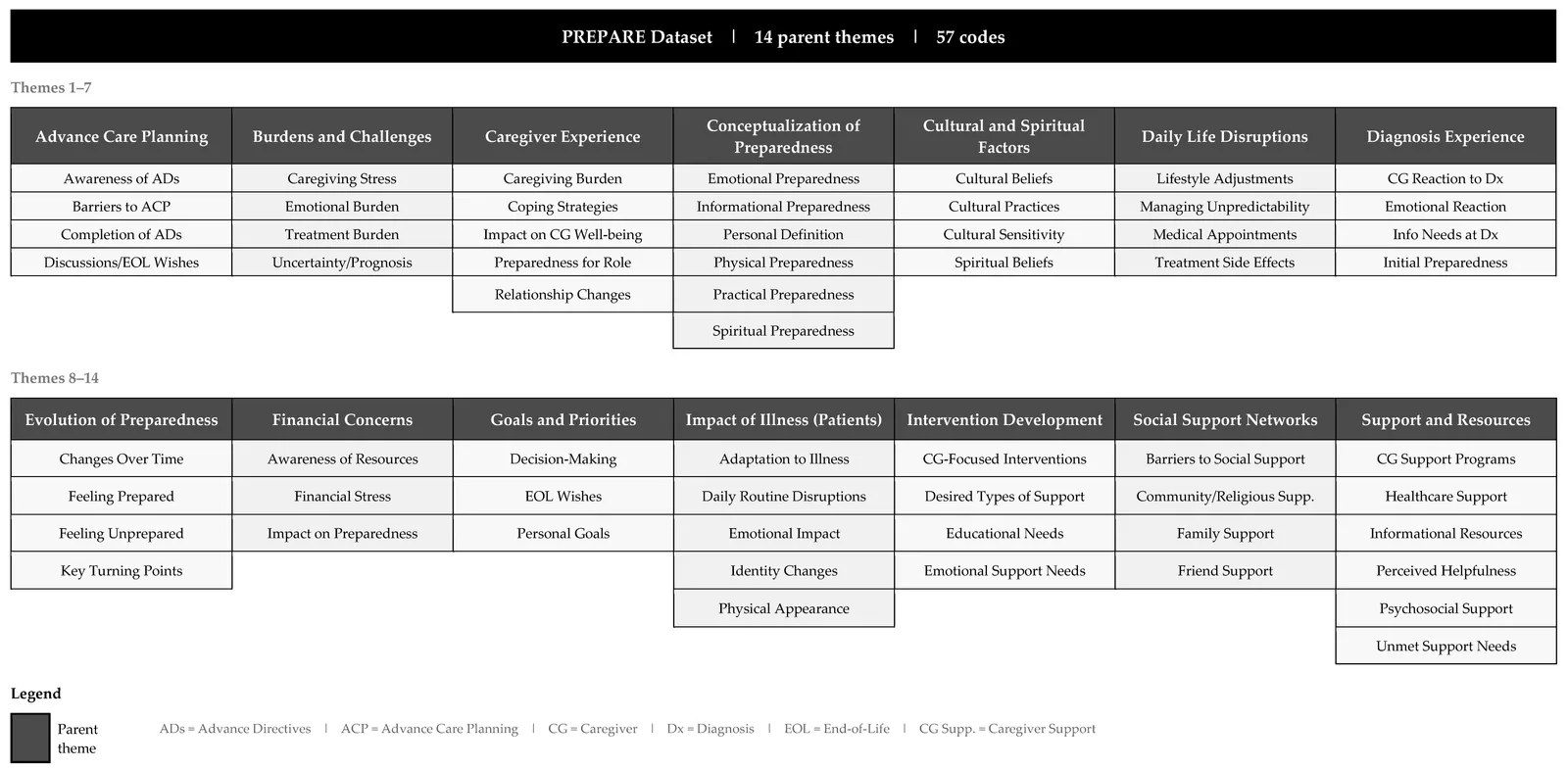
Coding tree.

Data were transcribed by members of the research team. Confidentiality measures were taken, such as the removal of all identifying information from transcripts prior to analysis, the assignment of participant identification codes in place of names, the storage of all audio recordings and transcripts on password-protected devices accessible only to approved research personnel, and the use of dyad number and role only in all reported excerpts. Data were analyzed using codebook thematic analysis, following Braun and Clarke [19,20]. The approach combines the flexibility of thematic analysis with the systematic rigor of a structured coding framework, making it well-suited to framework-guided qualitative inquiry. An initial codebook was developed deductively from the components of the Dyadic Cancer Outcomes Framework and the existing preparedness literature and refined inductively through line-by-line reading of a subset of transcripts. Although the initial codebook was developed deductively from the components of the Dyadic Cancer Outcomes Framework, the analytic process was explicitly deductive-inductive: codes and themes were continuously refined through iterative, line-by-line reading of participant transcripts, ensuring that the final categories reflected both the conceptual structure of the framework and the language, meaning, and experiences expressed by participants themselves across interviews (Figure 1). All transcripts were coded in NVivo 15, with both patient and caregiver transcripts coded using the same codebook to allow for dyadic comparison. Themes were defined and refined through team discussion, drawing on collective research experience, with attention to how findings mapped onto and extended the framework’s components. To ensure inter-rater reliability, 100% of the transcripts were independently coded by three members (L.R.R., C.Q.B., and G.L.E.) of the research team. Discrepancies were resolved through discussion and consensus. Data or results were not returned to participants for validation.

### 2.6. Ethical Considerations

This study was approved by the Institutional Review Board of Ponce Health Sciences University (Protocol #2411225855; approved and active). All participants provided informed consent prior to participation. Consent procedures were conducted in Spanish. Participants were informed of their right to withdraw at any time and to access psychosocial support through PAPSI if needed. All data were de-identified and assigned study identification numbers. Participant attributions use dyad number and role only (e.g., Patient, Dyad 001).

## 3. Results

A total of 11 patient–caregiver dyads participated in this study (n = 22) (Table 1). Patients were Hispanic women with advanced breast cancer, and caregivers were primarily female family members, including mothers, daughters, husbands, and siblings, reflecting the centrality of familial caregiving in this population. Results are organized according to the components of the Dyadic Cancer Outcomes Framework [17]: participant characteristics, relationship processes, individual and relational outcomes, the cancer care trajectory, and the social context shaping preparedness experiences.

### 3.1. Participant Characteristics: Understanding the Dyads Entering This Experience

Individual and dyadic characteristics, including cultural and spiritual identity, prior illness experiences, and financial context, shaped the lens through which participants understood and approached preparedness before and throughout the illness trajectory.

#### 3.1.1. Faith and Spirituality as a Foundational Orientation

Across virtually all dyads, faith was not a peripheral coping resource but a foundational framework through which participants understood illness, suffering, and what it meant to be prepared. Both patients and caregivers consistently invoked God, prayer, and the Holy Spirit as primary sources of strength, readiness, and meaning. One caregiver attributed her sense of preparedness directly to her spiritual life:

“*I am well prepared, you see, in life, in the blows that life gives you… That is what has made me feel strong. And they ask me, how can you do it? How do you manage? And I say, “The Holy Spirit.”*”

(Caregiver, Dyad 001)

This framing was not simply about coping after the fact; spiritual belief constituted a proactive preparedness resource. As a patient in the same dyad described, “One cannot be prepared. One must be guided by the will of God” (Patient, Dyad 001). This tension between human unpreparedness and divine readiness recurred across dyads, suggesting that for many participants, ultimate preparedness was understood as a relational orientation toward God rather than an individually achievable state.

#### 3.1.2. Financial Context as a Preparedness Barrier

Financial vulnerability was a pervasive characteristic that constrained participants’ capacity to prepare across multiple themes. Patients described having to choose between basic necessities and medical expenses:

“*Many times I could not afford the medications at the pharmacy. I would get them on credit. Or there were times when to pay for medications I could not pay for water or electricity. I fell behind on utilities to be able to pay for the medications.*”

(Patient, Dyad 001)

Caregivers experienced parallel financial pressures, often missing work without formal support. One caregiver remarked: “*I was absent from work a great deal… I even thought they were going to fire me for so many absences*” (Caregiver, Dyad 010). For many patients, the inability to work during treatment compounded financial stress significantly, with one patient describing the dual loss of income and professional identity as “*very jarring, the financial part… my work, I liked my work. I liked what I did and so it was like many things at once*” (Patient, Dyad 006).

### 3.2. Stressors and Demands: The Weight of Advancing Illness

Dyads navigated a constellation of stressors that evolved across the illness trajectory. Emotional burden, treatment demands, and prognostic uncertainty collectively defined the context within which preparedness was, or was not, achieved.

#### 3.2.1. Emotional Burden: The Most Pervasive Stressor

Emotional burden was the most heavily referenced finding across the entire dataset (19 sources, 118 references), underscoring that psychological distress was the central experience of advancing illness for this population. For caregivers, the emotional burden was compounded by witnessing suffering they felt powerless to alleviate. One mother described her daughter’s first chemotherapy night:

“*That first night was very hard… I felt so powerless. I could not do anything; even running a hand over her bothered her. And I felt powerless, even to pray.*”

(Caregiver, Dyad 002)

This dyadic mirroring, where the caregiver’s distress was directly shaped by witnessing the patient’s suffering, was a consistent pattern across dyads, demonstrating the fundamentally interdependent nature of the emotional burden.

#### 3.2.2. Treatment Burden and Daily Life Disruptions

Treatment burden was extensively documented (17 sources, 74 references), encompassing the physical demands of chemotherapy, radiation, hospitalizations, and surgical complications. Illness also disrupted fundamental aspects of daily life and identity. One patient described the loss of social life and prior routine:

“*It changed my life greatly, my daily routine. Because I was a person who was always out, sharing with friends. I would go out… And since then, I stopped going out.*”

(Patient, Dyad 001)

#### 3.2.3. Uncertainty and Prognosis

Living with prognostic uncertainty was a defining feature of the illness trajectory. One patient articulated this directly:

“*One is never prepared. Sometimes you go with the certainty that everything is fine, and when you go through this process, the doctor tells you you have to do this, and this other thing… One is never prepared.*”

(Patient, Dyad 001)

### 3.3. Relationship Processes: How Dyads Navigated Together

Relationship processes, the ways patients and caregivers supported, communicated with, and relied upon each other, were central mediators of preparedness. These processes unfolded within a cultural context of *familismo*, in which family networks constituted the primary support infrastructure for both patients and caregivers.

#### 3.3.1. Social Support: Family as the Primary Resource

Family support was the most extensively coded relational resource (19 sources, 64 references). Both patients and caregivers described family presence as essential to emotional survival. One patient captured the sustaining power of family:

“*The love of your children, the love of your siblings, that they call you, that they seek you out, that they look out for you. That helps you greatly. Family coming to you, close friends calling, all of that greatly contributes to a person’s recovery.*”

(Patient, Dyad 001)

Her caregiver reflected the same relational orientation: “*I mostly take refuge in talking a lot with her. We are always there supporting each other*” (Caregiver, Dyad 001). This mutual reliance, patient and caregiver as reciprocal sources of support, exemplifies the dyadic interdependence that characterized preparedness in this population.

#### 3.3.2. Communication and Shared Decision-Making

Communication patterns and shared decision-making processes (16 sources, 44 references) shaped how dyads confronted illness-related challenges. Some dyads demonstrated clear, proactive planning encompassing EOL arrangements, while others had not yet engaged these conversations. One patient described proactively settling her finances and communicating her wishes:

“*I told them: if I die, you know this house is supposed to be paid off… I told them things, because later one never knows.*”

(Patient, Dyad 006)

Her caregiver echoed this pattern:

“*We are there with our feet on the ground. That part of the situation. What cancer is. Her wishes.*”

(Caregiver, Dyad 004)

By contrast, other dyads had not yet engaged explicit EOL discussions:

“*No, actually these days I was talking with my mom about that. Because really, at any moment anything can happen.*”

(Patient, Dyad 009)

### 3.4. Conceptualizing Preparedness: A Multidimensional Experience

The central theoretical contribution of this study lies in how participants themselves conceptualized preparedness. Rather than describing it as a single state of readiness, participants articulated preparedness as multidimensional, personally defined, and evolving. Six distinct themes were identified from the data.

#### 3.4.1. Psychological and Emotional Preparedness

Psychological and emotional preparedness, a felt sense of emotional readiness, acceptance, and capacity to manage fear, was the most broadly referenced theme. For most participants, achieving this state required active internal work: controlling the mind, accepting the illness, and cultivating the will to live. One caregiver described her approach:

“*Take things calmly and have a lot of faith. Prayer is the most important. And not falling into that abyss of feeling, controlling the mind. Because the worst thing you can have is your mind out of control. Once you control your feelings, your emotions, and you have that control in your mind, you have everything.*”

(Caregiver, Dyad 001)

For patients, psychological preparedness often identified through a deliberate turning point, a moment of choosing life. One patient described her transformation while hospitalized:

“*And that day I managed to look forward… I held onto my faith and asked God: help me. I want to be with my grandchildren. I want to keep moving forward. And that is what happened.*”

(Patient, Dyad 001)

#### 3.4.2. Spiritual Preparedness

Spiritual preparedness was the most universally present theme across the sample and was distinctly different from psychological preparedness in that it was explicitly relational with God rather than individually enacted. One patient described faith as the mechanism through which she achieved readiness:

“*I asked God to give me the strength to be able to prepare myself and move forward. And that is what helped me prepare.*”

(Patient, Dyad 001)

A caregiver described spending the entire day of her daughter’s diagnosis in prayer as her primary preparatory act:

“*When she went to get her diagnosis, I spent that entire day praying: Lord, if it is your will, give me peace and help me, give me peace and strength, give me peace and fortitude, help me help her. And I tell you, I accomplished my purpose.*”

(Caregiver, Dyad 002)

Importantly, spiritual preparedness co-existed with fear and helplessness. Even participants who described themselves as spiritually prepared experienced moments where prayer itself felt out of reach, suggesting that spiritual preparedness is not a stable achievement but an ongoing practice requiring continual renewal.

#### 3.4.3. Informational Preparedness

Informational preparedness, having sufficient understanding of the disease, its trajectory, and what to expect, was identified as a significant unmet need. Participants consistently described receiving fragmented information from providers. One caregiver expressed a clear preference for transparent, anticipatory communication:

“*They should always speak honestly. To say: look, this is going to happen, this is going to happen. A very thorough orientation.*”

(Caregiver, Dyad 003)

A patient described the gap between clinical information and the experiential knowledge she needed:

“*The doctor tells me about what he does. But that process of being at home… I had two jobs. My life was based on full-time work, and finding myself sitting at home doing nothing has been a bit traumatic.*”

(Patient, Dyad 003)

#### 3.4.4. Practical Preparedness

Practical preparedness encompassed planning for logistics, legal arrangements, financial organization, and caregiving infrastructure. One caregiver proactively reorganized her home and purchased adaptive equipment upon her daughter’s diagnosis:

“*How to be prepared? Perhaps having the house set up with all the safety features or spaces to be able to care for the person. When she was diagnosed I bought one of those adjustable position beds.*”

(Caregiver, Dyad 002)

Advance care planning completion was variable across dyads. Importantly, the absence of formal legal documents did not always reflect unpreparedness; several participants had clear, communicated plans that were not legally formalized.

#### 3.4.5. Physical Preparedness

Physical preparedness involved readiness for bodily changes: treatment side effects, loss of physical function, and changes to appearance. One patient described the unanticipated weight of physical changes:

“*I wasted away. The treatment gave me severe diarrhea, vomiting, nausea… I lost all my hair. Many things.*”

(Patient, Dyad 001)

Caregivers also faced their own form of physical preparedness. One caregiver described the emotional labor of bathing her daughter after mastectomy:

“*That first time I thought I was prepared, and I was bathing her without her breasts, and I struggled not to look at her so she would not realize that I could not bear it. I told myself: I am prepared, I can look at her. That first time was challenging.*”

(Caregiver, Dyad 002)

Her gradual adaptation over the following months illustrates that physical preparedness is an acquired, evolving capacity rather than a fixed state.

#### 3.4.6. Caregiver Role Preparedness

Caregiver role preparedness, the caregiver’s sense of readiness to assume and sustain the caregiving role, was shaped by prior experiences and the near-total absence of formal preparation. One caregiver’s account captured the gap between perceived readiness and lived reality:

“*I understood that I was prepared to deal with whatever. But that night, when she spent the entire night screaming in pain, in anxiety… I felt so powerless.*”

(Caregiver, Dyad 002)

Another caregiver voiced the need for formal preparation that was never provided:

“*How do you deal with situations that arise when you do not have any education, any formal preparation to handle that?*”

(Caregiver, Dyad 002)

The absence of caregiver-specific preparation was consistent across dyads.

### 3.5. The Evolution of Preparedness: A Dynamic, Turning-Point-Driven Process

A defining characteristic of preparedness in this population was its dynamic, non-linear quality. Changes over time were referenced by 20 of 22 participants, confirming that preparedness is not a fixed state but an ongoing process shaped by key turning points across the illness trajectory.

#### 3.5.1. Unpreparedness at Diagnosis as a Universal Starting Point

The moment of diagnosis was consistently described as a point of acute unpreparedness. One patient described her reaction:

“*She told me: it came back positive for cancer. I froze, like loading… They just told me I have cancer, give me a break, let me process it.*”

(Patient, Dyad 002)

Caregivers described parallel shock:

“*I was already on my knees: Lord, give me strength, because this does not sound good… and when she arrived, all she did was fall into my arms and started to cry.*”

(Caregiver, Dyad 002)

#### 3.5.2. Key Turning Points That Reshaped Preparedness

Beyond diagnosis, several illness events served as key turning points requiring dyads to recalibrate their sense of preparedness: first chemotherapy, hospitalizations, surgical complications, and disease progression. One patient described preparedness as emerging through lived experience:

“*Everything is experience. The first time you go through something, you do not absorb it or accept it. But after, when you face it a second time, you already know more or less what you are going to go through and what to do.*”

(Patient, Dyad 001)

For caregivers, increased comfort with the demands of care also evolved gradually:

“*I would say that after a year and a half, that is when I felt a bit more comfortable dealing with her care.*”

(Caregiver, Dyad 002)

### 3.6. Social Context and Outcomes: Support Landscape and Unmet Needs

Participants identified both facilitators that supported preparedness and significant gaps in available support, particularly for caregivers and for the dyad as a relational unit. Family and faith communities were the most consistently cited facilitators. Peer support groups were described as transformative by those who had accessed them:

“*I learned so much. It is something many people dismiss, but you talk with others and they unburden themselves, and you unburden yourself… I did not treat cancer as a disease. I treated it as a purpose.*”

(Patient, Dyad 008)

Unmet support needs were extensive (18 sources, 36 references) and were particularly acute for caregivers. One caregiver stated plainly:

“*As a caregiver, no. I have not had support. I have not had a support network. Though I have had support from my family.*”

(Caregiver, Dyad 002)

Another described the absence of institutional acknowledgment:

“*I was absent from work a great deal and when I arrived at work and they asked me about it, I would cry, because at no point did they inform us, give us that support.*”

(Caregiver, Dyad 010)

The absence of dyad-level support was also named explicitly:

“*I understand that a key part of diagnosis and treatment is the support of immediate family. And I wish I had had them by my side when I had to go through that entire process. But they were not allowed in.*”

(Patient, Dyad 011)

## 4. Discussion

This study set out to conceptualize end-of-life (EOL) preparedness among Hispanic advanced breast cancer patient–caregiver dyads, a population for whom preparedness has been insufficiently studied and for whom existing conceptual frameworks have fallen short. Drawing on qualitative data from 11 dyads and guided by the Dyadic Cancer Outcomes Framework [17], our findings reveal EOL preparedness to be a multidimensional, relational, spiritually anchored, and dynamically evolving process that cannot be adequately understood or addressed at the individual level alone. Across the framework’s components, patient and caregiver characteristics, relationship processes, individual and relational outcomes, the cancer care trajectory, and the broader social context, a consistent pattern was identified: preparedness was not a state that participants achieved at a single point in time but an ongoing adaptive process shaped simultaneously by who they were, what they faced, how they navigated illness together, and what support was or was not available to them.

One of the most salient findings was the primacy of spirituality in participants’ preparedness frameworks. Faith was not described as one coping strategy among many but as the organizing principle through which all other themes of preparedness were understood and enacted. This is consistent with existing literature documenting the central role of faith as a source of meaning, hope, and resilience among Hispanic cancer patients and caregivers [21,22]. However, our findings extend this literature in an important direction: spirituality did not simply buffer the impact of unpreparedness but provided a framework within which unpreparedness itself was reinterpreted as an appropriate human condition. This distinguishes the preparedness experience of Hispanic dyads from conceptualizations in the broader EOL literature, where spirituality is often positioned as one domain among many, in that spirituality appeared as an overarching framework structuring all other themes.

Our findings also provide strong support for a dyadic intervention to EOL preparedness. Patients and caregivers did not experience preparedness independently. Their states of readiness were interdependent, with each partner’s capacity to cope directly shaping and being shaped by the other’s. Witnessing the patient’s suffering constituted the caregiver’s emotional burden; the patient’s sense of readiness was shaped by whether her family felt prepared; and the patient’s practical preparedness was enacted partly as an act of relational protection to spare family members from burden. This pattern aligns with the core propositions of the Dyadic Cancer Outcomes Framework [17], which posits that individual outcomes are mediated by relational processes and that the dyad is the appropriate unit of analysis in cancer care research. Notably, explicit discussion of relational dynamics was less prominent than other themes, suggesting that patients and caregivers often described the relational themes of preparedness indirectly, through accounts of family support, shared decisions, and mutual coping, rather than naming the relationship itself as a site of preparedness work. This may reflect a cultural pattern in which relational roles are enacted implicitly within familial structures rather than made objects of explicit reflection [23], implicating the need for interventions that incorporate: (1) joint dyadic delivery formats addressing both patient and caregiver simultaneously rather than in isolation; (2) spiritually integrated content that honors faith, prayer, and trust in divine will as legitimate and central preparedness practices; (3) dedicated caregiver-specific modules addressing role readiness, emotional regulation, and the management of patient distress, given that caregivers in this study received virtually no formal support as care recipients in their own right; (4) financial navigation components that address the structural burden of financial toxicity, which constrain preparedness across all themes simultaneously; (5) anticipatory and honest prognostic communication that prepares dyads for key illness turning points rather than leaving them to adapt reactively; and (6) culturally adapted advance care planning content that honors informal, family-based EOL planning consistent with *familismo*, alongside formal documentation. Together, these components constitute the empirical foundation for PREPARE, a culturally tailored, spiritually integrated, and dyadically delivered EOL preparedness intervention for Hispanic advanced breast cancer patient–caregiver dyads.

Financial stress was identified as a prevalent structural barrier to preparedness. Participants described choosing between utilities and medications, losing income during treatment, and accumulating debt, pressures that constrained informational, practical, emotional, and caregiver role preparedness simultaneously. This finding situates EOL unpreparedness within the broader context of health disparities facing Hispanic communities: Hispanic cancer patients face disproportionate financial toxicity that negatively impacts quality of life and treatment adherence [24]. Our findings extend this literature by demonstrating that financial vulnerability is also a preparedness issue, one that constrains the dyad’s capacity to engage with EOL planning at every level simultaneously.

The near-universal finding that preparedness evolved over time, shaped by key turning points including diagnosis, first chemotherapy, surgical complications, and disease progression, underscores its fundamentally dynamic nature [10,11,14]. Participants consistently described feeling unprepared at each new turning point, even when they had achieved a sense of readiness at earlier stages. Preparedness is not achieved once but rebuilt continuously, and interventions designed to support it must be longitudinal and responsive rather than delivered as a single episode of care.

A consistent finding was the invisibility of the caregiver within formal care systems. Most caregivers received no systematic support, psychoeducation, or recognition as care recipients in their own right, attending medical appointments, managing side effects, and responding to emotional crises without guidance or acknowledgment. This aligns with the literature on caregiver burden and unmet needs in cancer care [25] and is particularly pronounced in Hispanic caregiving contexts where *familismo* may create strong internal pressure to prioritize the patient’s needs above one’s own, turning cultural strength into structural vulnerability [23].

Results regarding advance care planning revealed a nuanced picture that challenges assumptions in standard ACP frameworks. While formal completion of advance directives was variable, many participants had engaged in informal, family-based EOL planning that was culturally coherent and functionally equivalent to formal documentation. Rather than interpreting this as avoidance or deficit, consistent with research on lower formal ACP rates in Hispanic populations [26], our findings suggest that this population engages in substantive EOL planning through relational and culturally embedded channels that existing frameworks do not adequately recognize or measure.

Taken together, these findings, the primacy of spirituality, the dyadic interdependence of patient and caregiver readiness, the structural weight of financial vulnerability, the dynamic and turning-point-driven nature of preparedness, the invisibility of caregivers in formal systems, and the cultural embeddedness of EOL planning, converge on a innovative conceptualization of preparedness that existing individual-level frameworks cannot capture. Existing definitions [15,16] conceptualize preparedness primarily as the caregiver’s sense of readiness to manage caregiving tasks. The present study makes several contributions with direct implications for caregiving improvement. By demonstrating that preparedness is co-constructed between patient and caregiver, it calls for dyadic rather than individual-level assessment and intervention. By establishing spirituality as the foundational organizing dimension rather than a supplementary domain, it challenges how EOL care is structured for Hispanic dyads. By situating caregiver invisibility within both cultural and structural analysis, it identifies a failure that requires not only clinical response but policy-level change. And by documenting the dynamic, turning-point-driven nature of preparedness, it provides an empirical argument for longitudinal, milestone-responsive intervention over single-episode models. These findings do not simply add a cultural modifier to existing frameworks; they require a reconceptualization of what EOL preparedness is, who it belongs to, and how it should be supported.

Several limitations should be acknowledged. The sample was drawn from a single geographic context in Puerto Rico, and the findings may not generalize to Hispanic dyads in other regions or with different national origins, acculturation levels, or healthcare system contexts. The qualitative cross-sectional design captures retrospective accounts at a single illness stage, precluding prospective documentation of how preparedness evolves in real time. Although the study emphasized dyadic processes, most interviews were conducted individually, which may have constrained the capture of jointly negotiated preparedness dynamics. Future research using joint dyadic interviews would strengthen this analysis. The relatively small sample size, while appropriate for qualitative inquiry, limits theoretical saturation for less-referenced codes, including Relationship Changes and Caregiver-Focused Interventions. Additionally, as only one primary informal caregiver per dyad was enrolled, the perspectives of secondary caregivers and other household members who may also be involved in caregiving were not captured. In Hispanic families shaped by *familismo*, caregiving is frequently a collective endeavor distributed across multiple family members, and future research using family systems or network-based approaches would provide a more complete picture of how caregiving responsibilities and preparedness experiences are shared across the household unit. An additional limitation is that the study does not address end-of-life care for persons without spiritual or religious beliefs. Finally, social desirability may have influenced accounts on sensitive topics such as EOL planning and caregiver burden. Future directions include validating the proposed definition through member-checking and expert review, developing a dyadic preparedness scale, and conducting longitudinal and comparative research across Hispanic subgroups.

## 5. Conclusions

This study presents the first qualitative, dyadic examination of EOL preparedness among Hispanic advanced breast cancer patient–caregiver dyads. The findings reveal that preparedness in this population is not a checklist of tasks completed or documents signed but a deeply human, relational, and spiritually grounded process of facing the unknown together. Six interdependent themes of preparedness were identified, organized within a dyadic relational structure and fundamentally shaped by cultural values, faith, family bonds, and financial vulnerability. Preparedness was dynamic and turning-point-driven, challenged anew at each stage of illness progression and rebuilt through spiritual resilience, family support, and experiential knowledge.

## Figures and Tables

**Table 1:** Sociodemographic and clinical characteristics of patient–caregiver dyads.

Variable	Patients (n = 11)	Caregivers (n = 11)
**Sex**		
Female	11 (100%)	5 (45%)
Male	0 (0%)	6 (55%)
Age, mean (range)	52 (34–70)	49 (21–72)
**Relation to patient**		
Husband		4 (36%)
Mother		2 (18%)
Sister		2 (18%)
Daughter		1 (9%)
Son		1 (9%)
Nephew		1 (9%)
**Marital status**		
Married	6 (55%)	6 (55%)
Single	3 (27%)	2 (18%)
Divorced	1 (9%)	0 (0%)
Widowed	1 (9%)	1 (9%)
Consensual union	0 (0%)	1 (9%)
Not reported	0 (0%)	1 (9%)
**Education**		
High school	2 (18%)	6 (55%)
Bachelor’s degree	6 (55%)	2 (18%)
Technical degree	1 (9%)	3 (27%)
Master’s/Doctorate	2 (18%)	0 (0%)
**Spirituality/Religion**		
Yes	11 (100%)	9 (82%)
No	0 (0%)	2 (18%)
Family history of breast cancer	10 (91%)	8 (73%)
**Clinical characteristics (patients only)**		
**Cancer stage**		
Stage III	9 (82%)	
Stage IV	2 (18%)	
**Year of diagnosis**		
2018–2021	4 (36%)	
2022–2025	7 (64%)	
**Treatments received**		
Radiotherapy	6 (55%)	
Chemotherapy	9 (82%)	
Surgery	11 (100%)	
Hormonal therapy	6 (55%)	

## Data Availability

The data presented in this study are available upon request from the corresponding author due to privacy.

## Abbreviations

The following abbreviations are used in this manuscript:

EOL	End-of-Life

## Appendix A. Dyadic Preparedness Semi-Structured Interview

**Introduction**

- Provide a warm and empathetic introduction, explaining the purpose of the interview to understand experiences with end-of-life readiness in advanced breast cancer.
- Ensure confidentiality and obtain informed consent.

**Preparation Components**
***Exploratory Questions***

- Reflecting on your journey so far, how do you define being “prepared” for the future stages of your disease?
- Can you expand on which aspects of preparation are most important to you (e.g., emotional, spiritual, physical, or informational)?

**Needs and Development of Interventions**
***Current Support and Needs***

- Have you been offered interventions or resources to help you prepare for the end of life? If so, how helpful were they? (Patients)
- Have you had access to caregiver support programs or resources? What was beneficial and what was missing? (Caregivers)

***Future Needs and Design of Interventions***

- Based on your experiences, what specific types of support or information do you think would be most helpful for others facing advanced breast cancer to feel prepared for the end of life? (Both)

**Experience and Preparation for the End of Life**
***Sharing Experiences***

- Can you recall a specific instance where you felt well-prepared or noticeably unprepared? What made the difference? (Both)

***Preparation Evolution***

- How do you think your sense of preparedness has evolved over time? Were there specific events or experiences that shaped this change? (Both)

**Individual Experiences (Patients)**
***Impact of the Disease***

- How has your illness influenced your sense of identity and daily routines?
- Can you share how you have adapted to the changes in your life due to your illness?

**Individual and Dyadic Experiences (Caregivers)**
***Impact of Care***

- How has the experience of caring affected your personal well-being and your relationship with your loved one?
- What coping strategies have you found most effective for managing caregiving stress?

**Burdens**
***Challenges***

- What aspects of your illness or treatment (for patients)/caregiver role (for caregivers) do you find most challenging?
- How do these burdens affect your sense of preparedness and overall well-being?

**Goals**
***Wishes and Priorities***

- Looking ahead, what are your most cherished goals and desires? (Both)
- How do you approach making decisions about your care and priorities? (Both)

**Cultural Factors**
***Beliefs and Practices***

- How do your cultural or spiritual beliefs influence your view of end-of-life preparation and care? (Both)
- Are there specific cultural or spiritual rituals or practices that you want to incorporate into your care plan? (Patients)
- Are there cultural or spiritual considerations that you feel health care providers should be more aware of or sensitive to in their care? (Caregivers)

**Additional Considerations**
***Social Support Network***

- Can you tell me a little bit about your social support network (e.g., family, friends, and religious community)? (Both)
- How does your social support network help you cope with the challenges of advanced breast cancer? (Patients)
- How does your social support network help or hinder you in your role as a caregiver? (Caregivers)

**Financial Considerations**

- Have you experienced any financial burdens related to your illness or caregiving role? (Both)
- If so, how have these financial concerns impacted your sense of end-of-life readiness? (Both)
- Are you aware of any resources that can help with the financial costs associated with end-of-life care? (Both)

**Advance Directives**
  Advance directives, such as living wills and durable power of attorney for health care, can help ensure that your wishes are followed at the end of life.

- Have you completed any advance directives? (Both)
  ○ If so, can you tell me a little bit about them? (Both)

**Topics**
***Initial Diagnosis (Reaction to Diagnosis)***

○ **Patients:** Can you guide me through the initial stages after receiving your diagnosis of advanced breast cancer? What were your thoughts and emotions? Did you feel prepared to hear this news, or were you taken by surprise? In light of this, is there anything you wish you had known or done differently to feel more prepared?
○ **Caregivers:** How did you learn about your loved one’s diagnosis? What were your initial thoughts and emotions? How did you feel about your readiness to support your loved one on this journey? Were there any resources or information you wish you had had access to at the time?

***Physical Appearance (Dealing with Changes in Physical Appearance Caused by Cancer Treatment)***

○ **Patients:** Cancer treatments can sometimes cause changes in physical appearance. How have you dealt with these changes, if any? How have these changes impacted your self-esteem or body image?
○ **Caregivers:** Has your loved one experienced changes in their physical appearance due to treatment? How have you supported them in coping with these changes? How have these changes affected your loved one’s emotional well-being? Did you feel prepared to help your loved one manage these changes?

***Social Support (Importance of Talking About the Diagnosis and Getting Support from Friends and Family)***

○ **Patients:** Who did you turn to for support after your diagnosis? How did talking about your illness with loved ones impact your well-being? Did you feel comfortable talking openly about your illness, or were there challenges? Did you feel like you had a strong support system, or were there people you wished you could have talked to more openly?
○ **Caregivers:** Who did you turn to for support after learning of your loved one’s diagnosis? How has your support network helped manage caregiving challenges? Did you feel ready to take on this role, or did you wish you had more information or support? How did your loved one feel about the level of support they have received?

***Disruptions in Daily Life (Disruptions Caused by Medical Appointments, Hospitalizations, and Side Effects)***

○ **Patients:** Can you describe how your daily life has been affected by medical appointments, hospitalizations, or side effects of treatment? How have you managed these disruptions and how have they affected your overall well-being? Did you feel prepared for these disruptions, or were there ways you wished you could have anticipated or managed them better?
○ **Caregivers:** How have your loved one’s medical appointments, hospitalizations, or side effects impacted your daily routine and responsibilities? How have you dealt with these disruptions? How have they affected your ability to provide care? Did you feel prepared to handle these disruptions, or were there aspects you wished you could have anticipated or handled better?
